# Nutritional Composition, Essential Oil Profiling, and Post-Digestion Phytochemical and Antioxidant Properties of Fennel Pignut (*Bunium ferulaceum*) Seeds

**DOI:** 10.3390/foods15193552

**Published:** 2026-10-06

**Authors:** Amirouche Deghima, Nadjat Righi, Elisabete Coelho, Maria Inês Arranca, Sónia S. Ferreira, Faiza Baali, Susana M. Cardoso, Diana Ansorena, Iciar Astiasaran, Manuel A. Coimbra

**Affiliations:** 1 Departamento de Ciencias de la Alimentación y Fisiología, Facultad de Farmacia y Nutrición, CIN Centro de Investigación en Nutrición, INS Instituto de Nutrición y Salud, Universidad de Navarra, Irunlarrea s/n, 31008 Pamplona, Spain; iastiasa@unav.es; 2 Laboratory of Genetics, Biotechnology and Valorization of Bioresources, University of Biskra, BP 145RP, Biskra 07000, Algeria; 3 Laboratory of Applied Biochemistry, Faculty of Natural and Life Sciences, University Ferhat Abbas Setif 1, Setif 19000, Algeria; n.righi34@gmail.com; 4 LAQV-REQUIMTE, Department of Chemistry, University of Aveiro, Campus Universitario de Santiago, 3810-193 Aveiro, Portugal; ecoelho@ua.pt (E.C.); inesarranca@ua.pt (M.I.A.); soniasferreira@ua.pt (S.S.F.); susanacardoso@ua.pt (S.M.C.); mac@ua.pt (M.A.C.); 5 Department of Biology, Faculty of Nature and Life Sciences and Earth Sciences, University of Ghardaia, BP 455, Ghardaïa 47000, Algeria; baali.faiza@univ-ghardaia.dz

**Keywords:** fennel pignut seeds, nutrients, polyphenols, in vitro digestion, fatty acids, minerals

## Abstract

Fennel pignut (*Bunium ferulaceum*) seeds are used in different foods as food ingredients, contributing to the flavor and aroma of various culinary preparations, yet they remain underexplored as a source of nutritional and bioactive compounds. This study aims to comprehensively characterize the nutritional and phytochemical matrix of *B. ferulaceum* seeds across their macronutrient, mineral, and phenolic fractions and track the phenolic compounds’ contents and shifts in a multi-assay antioxidant capacity across simulated oral, gastric, and intestinal digestion. The results revealed a carbohydrate-dominant seed (39%), with uronic acid, glucose, and mannose as the principal constituents, and a lipid fraction (4%) characterized by oleic/petroselinic acids (54%). Proteins (19%) provided a nutritionally complete amino acid profile, with arginine as the predominant residue. Phytochemical analysis identified β-caryophyllene and germacrene-B in the essential oil fraction, while the polar extract was rich in hydroxycinnamic derivatives (caffeoylquinic and di-caffeoylquinic acids) and quercetin glycosides. Undigested seed extract contained a total polyphenol content (TPC) of 75.08 ± 1.91 µg gallic acid equivalent (GAE)/mg extract with a good 2,2′-azino-bis(3-ethylbenzothiazoline-6-sulphonic acid) (ABTS) radical-scavenging ability of 340 μg TE/mg extract. Simulated digestion reduced polyphenol content by 10% during intestinal phase, while antioxidant activity dropped by 82% [2,2-diphenyl-1-picrylhydrazyl (DPPH) radical] and by 46% for oxygen radical absorbance capacity (ORAC) during the same phase compared to the undigested sample. Organic acids and caffeoylquinic derivatives remained detectable during in vitro simulated digestion compared to flavonoid glycosides such as quercetin derivatives, which were undetectable after intestinal digestion. Fennel pignut seeds represent a nutritionally dense and phytochemically rich ingredient. The seeds retained substantial antioxidant activity following vitro digestion, supporting their incorporation into functional food formulations.

## 1. Introduction

In recent years, the interest among consumers in choosing healthy foods as part of their dietary pattern has increased [1]. Consumers desire functional foods, especially nuts, seeds, fruits, and vegetables, as part of healthy lifestyles to maintain good health [1]. With the functional food industry witnessing substantial growth in both research and market trends, it was estimated that the global revenue of the functional foods market was valued at approximately USD 441.56 billion [2]. As functional foods are gaining more demand, there has been consistent exploration of newer food constituents enriched with nutritional and bioactive components [2]. In this context, seeds have played important roles in human nutrition and health since ancient times. In addition, seeds have now been widely consumed as healthy snacks or incorporated into daily diet as cakes, cookies, cereal bars, and pastries to improve their nutritional content and functional properties [1,3]. Seeds are an important source of dietary fiber, essential fatty acids, and antioxidants, and they contribute to decreasing the risk of chronic diseases, including cardiovascular disorders, diabetes, and certain types of cancer. Thus, considering seeds an integral part of the diet is a crucial step towards enhancing nutrition and health [3].

The *Apiaceae* family has the largest number of different seed spices that are widely recognized for their nutritional, medicinal, and industrial significance, owing to their rich composition of bioactive compounds [4]. Notable examples include cumin, coriander, and caraway, which not only enhance food palatability but also exhibit remarkable therapeutic properties, including antifungal, antibacterial, anticancer, and antidiabetic effects, primarily attributed to their high antioxidant content [5]. Additionally, seeds from the *Apiaceae* family are traditionally employed in the treatment of digestive and endocrine disorders, demonstrating hypoglycemic, hypolipidemic, and hepatoprotective activities, which reinforce their potential applications in functional foods, pharmaceuticals, and cosmetics [6]. The *Apiaceae* family is also distinguished by its production of specialized metabolites, including volatile phenylpropanoids, furanocoumarins, sesquiterpene coumarins, polyacetylenes, and phthalides, many of which are unique to this botanical group and present significant opportunities for bioprospecting [7]. On an industrial scale, these seeds are primarily targeted for essential oil extraction, yet their non-volatile lipid fraction and polar fraction rich in valuable nutraceutical and bioactive compounds remain underexplored and are often discarded as waste [6,7,8].

*Bunium ferulaceum* (Fennel Pignut) is a multipurpose food plant whose aerial parts are used for food flavoring [9,10] and for their antioxidant and anti-inflammatory properties [9]. Its tubers are known as an excellent food source due to their high nutritional density [11,12,13,14,15,16]. While the seeds of *B. ferulaceum* (BFS) are used as food ingredients, contributing to the flavor and aroma of various culinary preparations [5], they remain underutilized as they are largely underexplored. The limited available studies are focused on their essential oil’s composition, their antibacterial activity [10,17] and on the composition of their polar extracts [18,19]. While previous studies have examined the volatile oils and some extractable phenolics of *Bunium ferulaceum* seeds, their broader nutritional architecture and the physiological fate of its bioactives remain unexamined. Simulated in vitro gastrointestinal digestion is essential in food research because raw measurements of crude extracts do not reflect the stability or physiological fate of polyphenols as they undergo pH shifts and enzymatic action during digestive transit [20] (Gayoso et al., 2018).

Building on this rationale, this study addresses two central objectives: We assess how the nutritional and phytochemical matrix of *B. ferulaceum* seeds is constituted across its carbohydrate, lipid, protein, mineral, and phenolic fractions, and evaluate the behavior of the phenolic compounds during simulated gastrointestinal digestion. In this study, incorporating an in vitro digestion model allowed us to track changes in total phenolic, flavonoid, and flavonol contents (TPC, TFC, and FOL spectrophotometrically), evaluate shifts in radical-scavenging ability and antioxidant capacity across complementary assays (DPPH, ABTS, and ORAC), and monitor the detectability of specific phenolic constituents across oral, gastric, and intestinal phases. Ultimately, this approach provides physiologically relevant evidence regarding the digestive stability and residual functional potential of *Bunium ferulaceum* seed bioactives beyond static, undigested extract analysis and evaluates the functional and nutraceutical potential of these underutilized seeds.

## 2. Materials and Methods

### 2.1. Chemicals

All chemicals and reagents were of analytical grade and purchased from Sigma-Aldrich Co. (Steinheim, Germany), unless otherwise specified. For the in vitro digestion assay, α-amylase from human saliva (A1031, 852 units/mg protein), pepsin from porcine gastric mucosa (P7000, 674 units/mg protein), pancreatin from porcine pancreas (P1750, 4 × United States Pharmacopeia specifications) and bile extract (B8631) were also purchased from Sigma-Aldrich Co. (Steinheim, Germany).

### 2.2. Plant Material

The seeds of *Bunium ferulaceum Sm*. (*Bunium incrassatum* (Boiss.) Amo.) were harvested from the Bordj Ghedir locality in the Bordj Bou Arreridj province (East of Algeria: 35°50′08.7″ North 4°58′19.0″ East, 1100 m above sea level) in June 2018. Professor Gharzouli from the Faculty of Nature and Life Sciences at the University of Setif (Setif, Algeria) and Professor Boujelal from the Faculty of Sciences at the University of M’sila (M’sila, Algeria) identified the plant material and a voucher specimen (AB-110) was deposited at the herbarium of the Faculty of Sciences, M’sila University (M’sila, Algeria). The seeds were sieved to clean them from dust and carefully separated from any other contaminants. Finally, they were left to dry in the dark at room temperature. The dried seeds were stored in air-sealed bottles at room temperature and powdered using an electric grinder before use.

### 2.3. Nutritional Composition

The nutritional composition of dried *Bunium ferulaceum* Sm. seeds was determined using standard AOAC methods [21]. Moisture content was measured using method 945.15, total fat by method 960.39, ash content by method 920.153, and total crude proteins by the macro-Kjeldahl method (N × 6.25) (method 928.08). The results of all nutritional composition analyses were expressed in reference to dry seed weight.

Total carbohydrates were quantified by a multi-step process involving acidic hydrolysis, reduction to alditols, acetylation, and gas chromatography with a flame ionization detector (GC-FID) (Perkin Elmer Clarus 400, Perkin Elmer, MA, USA). Neutral sugars were initially hydrolyzed with 12 M H_2_SO_4_ for 3 h at room temperature; then, hydrolysis continued for 2.5 h at 100 °C. The resulting hydrolysate was reduced using NaBH_4_ (15% *w/v* in 3 M NH_3_) for 1 h at 30 °C and acetylated with acetic anhydride and 1-methylimidazole as a catalyst for 30 min at 30 °C. The resulting alditol acetates were analyzed by GC-FID using 2-deoxyglucose as an internal standard. Uronic acids were determined using the *m*-phenylphenol colorimetric method with galacturonic acid (GalA) as a standard, following the procedure described by Fernandes et al. [22].

To isolate polysaccharides, ethanol-soluble compounds (phenolics and free sugars) were removed from 8 g of *Bunium ferulaceum* seeds by maceration in 80 mL of 80% ethanol for 10 min. The mixture was then centrifuged, and the ethanol-soluble fraction (ESF), representing 14.8% of the dry matter, was separated from the remaining ethanol-insoluble fraction (EIF). The EIF was further boiled in distilled water, allowing us to obtain a water-soluble fraction (WSF) that was separated from the water-insoluble fraction (WIF) [23].

The mineral content analysis was performed on the incineration residue obtained in Section 2.3 using atomic absorption spectroscopy (AAS) (Perkin-Elmer A Analyst 800 spectrometer, Perkin Elmer, Waltham, MA, USA). The incineration residue was digested with concentrated hydrochloric acid and diluted with distilled water before analysis as previously described [24].

The fatty acid profile was determined in the extracted lipid fraction according to the method of Ansorena et al. [25], described in the work of Deghima et al. [24]. Fatty acids were converted into their corresponding fatty acid methyl esters (FAMEs) by transesterification using boron trifluoride in methanol according to AOAC Official Method 969.33 [26]. Chromatographic analysis was performed using a Perkin-Elmer Autosystem XL gas chromatograph equipped with a flame ionization detector (FID) (Clarus 500, PerkinElmer, Waltham, MA, USA) and a high-polarity capillary column SP™-2560 (100 m × 0.25 mm internal diameter, 0.2 µm film thickness; Sigma-Aldrich, St. Louis, MO, USA). The operational temperatures were set to 250 °C for the injection port and 260 °C for the FID detector. The oven temperature program was initiated at 175 °C for 10 min, increased to 200 °C at a rate of 10 °C/min, and finally ramped to 220 °C at 4 °C/min, where it was maintained isothermally for 15 min. Hydrogen was used as the carrier gas at a head pressure of 20.5 psi, with a split flow rate of 120 cm/s.

The separation of FAMEs was achieved based on differences in fatty acid chain length, and degree of unsaturation, through differential polar interaction with the stationary phase, producing distinct retention times. Identification of FAMEs was performed by comparing sample retention times with those of pure individual methylated standards (Sigma-Aldrich, St. Louis, MO, USA) covering saturated (SFA), monounsaturated (MUFA), cis-polyunsaturated (PUFA), and specific trans isomers. Unequivocal peak identification and separation from neighboring compounds (including eicosapentaenoic acid [EPA, C20:5] and eicosadienoic acid [C20:2]) were further confirmed by individually spiking sample matrices with pure reference standards. Quantification of individual fatty acids was performed using the internal standard method, adding heptadecanoic acid methyl ester (C17:0 ME, Sigma-Aldrich) prior to methylation. Assuming equal response factors (1.0) for all FAMEs, relative peak areas were used to calculate the individual fatty acid proportions, and the results were expressed as a percentage of the total fatty acid content (g fatty acid/100 g total fatty acids).

The results obtained were used to calculate the index of atherogenicity (IA), the index of thrombogenicity (IT) and the hypocholesterolemic/hypercholesterolemic ratio h/H [24] using the following equations:

IA = (C12:0 + 4C14:0 + C16:0)/(∑PUFAω6 + ∑PUFA ω3 + ∑MUFA)

IT = (C14:0 + C16:0 + C18:0)/(0.5 (MUFA + PUFA ω6 + ω3 × PUFA ω3) + (PUFA ω3/PUFAω6))

h/H = (C18:1 + C18:2n-6 + C20:4n-6 + C18:3n-3 + C20:5n-3 + C22:5n-3 + C22:6n-3)/(C14:0 + C16:0).

In this equation, for quantitative purposes, oleic acid (18:1n-9, cis-Δ9) and petroselinic acid (18:1n-12, cis-Δ6) are both included in the C18:1 value.

To determine the amino acid profile from *B. ferulaceum* seeds, proteins were extracted using alkaline extraction at pH = 10, followed by acid precipitation at pH = 4. The resulting protein-rich fraction was neutralized, frozen (−80 °C), and then freeze-dried. The extraction yield and recovery rate were calculated as follows:

Extraction Yield (%) = (m1/m2) × 100

Recovery rate (%) = [(m1 × P1)/(m2 × P2)] × 100

m1: weight of the protein powder, m2: weight of the defatted seeds powder, P1: protein content of the protein powder, and P2: protein content of the defatted seed powder.

The amino acid profile of the protein-rich fraction determination was performed using the Waters™ Kairos™ Amino Acid Kit (Low Throughput), coupled with an ACQUITY UPLC-H Class system (Waters Corporation, Milford, MA, USA) interfaced with a QTRAP 5500 mass spectrometer (SCIEX, Darmstadt, Germany). Sample preparation consisted of acid hydrolysis in which approximately 100 mg of sample was weighed to the nearest milligram, placed in a screw-capped tube with 5 mL distilled water and 5 mL diluted hydrochloric acid (37.5% HCl, 1:1 dilution giving a final hydrolysis concentration of approximately 6 M HCl), sealed with Pyrex stoppers, and maintained at 105 °C for a minimum of 8 h. After cooling to room temperature, the hydrolysate was quantitatively transferred to a 100 mL beaker and rinsed with distilled water, and the pH was adjusted to 6–7 using sodium hydroxide. The final volume was brought to 50 mL in a volumetric flask using distilled water. For protein precipitation, 10 µL of the hydrolysate (or of the reconstituted calibrator) was combined with 10 µL internal standard (prepared in 10% sulfosalicylic acid), vortexed for 5 s, and 180 µL methanol containing 0.1% formic acid was added. Following vortexing for an additional 5 s, high-concentration samples were diluted with 0.1 M HCl as needed, and the samples were centrifuged at 9000× *g* for 10 min. Calibrators were reconstituted in 0.1 M HCl, vortexed for 10 s, and roller-mixed for 30 min prior to use. Amino acid derivatization was performed by adding 70 µL borate buffer (prepared by adding 430 µL of 0.5 M NaOH to the kit’s borate buffer vial), 10 µL supernatant from the precipitation step, and 20 µL AccQ•Tag reagent (previously heated at 55 °C for 10 min to ensure complete dissolution). The mixture was vortexed for 5 s, allowed to stand at room temperature for 1 min, and then heated at 55 °C for 10 min. Chromatographic separation was accomplished using a CORTECS UPLC C18 column (1.6 µm, 2.1 × 150 mm) maintained at 55 °C, with a mobile phase consisting of water with 0.1% formic acid (mobile phase A) and acetonitrile with 0.1% formic acid (mobile phase B), at a flow rate of 0.5 mL/min. The gradient profile was as follows: 0–1.0 min at 99% A/1% B, 1.0–2.0 min ramping to 87% A/13% B, 2.0–5.5 min reaching 85% A/15% B, 5.5–6.5 min transitioning to 5% A/95% B, 6.5–7.5 min held at 5% A/95% B, 7.5–7.6 min returning to 99% A/1% B, and 7.6–9.0 min held at 99% A/1% B. The injection volume was 1 µL of the derivatized sample, and the total analysis time was 9 min. Mass spectrometry detection was conducted in positive electrospray ionization (ESI+) mode with a spray voltage of 5500 V, ion source gas 1 at 55 psi, ion source gas 2 at 55 psi, curtain gas at 28 psi, collision-activated dissociation gas at level 6, and a source temperature of 550 °C. Glutamine and asparagine were not reported separately, as acid hydrolysis converts both into glutamic acid and aspartic acid via deamidation. The reported glutamic acid/glutamine and aspartic acid/asparagine values therefore represent both forms, not their individual free, pre-hydrolysis forms.

### 2.4. Essential Oil Extraction and Characterization

Essential oils were extracted from the seeds of *B. ferulaceum* using a Clevenger apparatus (Glassco, Barcelona, Spain). One hundred grams of seeds were subjected to hydrodistillation in one liter of water for 3 h. The extracted essential oil was collected and stored in dark containers at 4 °C [27].

The obtained essential oils were analyzed using an Agilent Technologies 6890N Network gas chromatograph (from Agilent Technologies, Inc., Santa Clara, CA, USA) equipped with a 30 m long, 0.32 mm internal diameter, and 0.25 µm film thickness DB-FFAP fused silica capillary column. Helium was used as the carrier gas at a flow rate of 1.7 mL per minute, with the column pressure maintained at 3.52 psi. The temperature program used for the analysis was as follows: the initial temperature was set at 60 °C, followed by a linear increase of 2 °C per minute up to 180 °C. The temperature then increased at a rate of 5 °C per minute until it reached 220 °C, where it was held constant until the end of the run (70 min). The gas chromatograph was connected to an Agilent 5973 mass quadrupole (from Agilent Technologies, Inc., USA) selective detector operating in electron impact mode at 70 electron volts. The detector scanned the mass range from 33 to 300 *m*/*z* units in a 1 s cycle in full scan mode. The injection was performed in splitless mode.

The essential oil was injected directly after dilution in dichloromethane (1:200). For quantification, 0.496 mg of the internal standard 3-octanol was added prior to analysis. The identification of volatile compounds was achieved by comparing the GC retention index and mass spectra. All mass spectra were also compared with the library data system of the GC-MS equipment (Wiley 275). The identified compounds were quantified based on the GC peak areas, using 3-octanol as the internal standard [27].

### 2.5. Extract Preparation

The extract was prepared from the ground seeds using the method detailed in our previous study [9]. Briefly, the ground seeds (100 g) were extracted using two-step maceration with hydromethanolic solutions of decreasing methanol concentration (85% followed by 50%) (1:10, *w*/*v*). Each step involved 24 h of stirring at room temperature, followed by filtration. The combined extracts were concentrated under reduced pressure using a rotary evaporator and finally dried in a ventilated oven at 40 °C [9].

### 2.6. In Vitro Gastrointestinal Digestion (GID) and Antioxidant Activity

The extract was subjected to an in vitro digestion process using the procedure of Gayoso et al. [20] adapted by Deghima et al. [24]. Samples (200 mg) of the dried extract were placed in different Falcon tubes (Sigma) and dissolved in 12 mL of distilled water yielding a starting extract concentration of 16.67 mg/mL. The tubes were maintained continuously at 37 °C in a water bath with continuous stirring. Three separate, independent tubes were used to represent the three sequential digestion stages (oral, gastric, and intestinal) without reducing the reaction volume by intermediate sampling.

Initially, the pH in all tubes was adjusted to 6.5 with 1 M sodium bicarbonate (NaHCO_3_) and oral digestion was initiated in the three tubes by adding 50 μL of α-amylase stock solution (1.3 mg/mL in 1 mM CaCl_2_; α-amylase from human saliva, Sigma A1031, 852 units/mg protein) for 2 min, yielding a final reaction volume of 12.05 mL, a final enzyme concentration of 5.39 µg/mL, and a final enzyme activity of 4.60 U/mL.

At the end of this step, the pH was adjusted to 2.5 using 3 M HCl, and a tube was immediately snap-frozen (–80 °C) (representing the oral digestion OD). A pepsin stock solution (66 μL, 160 mg/mL in 0.1 M HCl; porcine gastric mucosa pepsin, Sigma P7000, 674 units/mg protein) was added to the remaining tubes to simulate gastric digestion for 2 h at 37 °C under continuous stirring. The resulting final pepsin concentration in the gastric reaction (12.12 mL final volume) was 872 µg/mL, corresponding to a final gastric pepsin activity of 587.4 U/mL. Gastric lipase was omitted in this protocol since the extract was prepared using hydro-methanolic mixtures (polar extract). At the end of the 2 h gastric phase, the pH was brought up to 7 using 1 M sodium bicarbonate to inactivate pepsin, and the second tube was immediately frozen at −80 °C, representing gastric digestion (OD and GD).

Intestinal digestion was initiated in the remaining tube by adding 500 μL of a 1:1 mixture of pancreatin (4 mg/mL; porcine pancreas pancreatin, Sigma P1750, 4xUSP specifications) and bile extract (25 mg/mL; Sigma B8631) for 2 h at 37 °C under continuous stirring, and the pH was then adjusted to 7.5 using 1 M sodium bicarbonate, yielding a final intestinal volume of 12.62 mL, with final concentrations of 79.3 µg/mL pancreatin and 495 µg/mL bile extract. Then, the tubes were snap-frozen at −80 °C until use. Subsequently, the three tubes were thawed (4 °C) and then centrifuged at 4000× *g* (A-4-62 Rotor, Model 5810R centrifuge, Eppendorf, Barcelona, Spain) for 40 min at 4 °C, and the supernatant, which is considered the bioaccessible fraction, was collected and lyophilized for further analysis. The supernatant from the first tube represents the bioaccessible fraction from oral digestion (OD), and that from the second tube represents the bioaccessible fraction from gastric digestion (OD and GD), while the supernatant from the third tube represents the bioaccessible fraction from intestinal digestion (ID, OD, and GD). For each digestion, three independent triplicates were performed (n = 3). Additionally, a blank digestion was also carried out, including all the digestive reagents, replacing the sample with water.

Following lyophilization, the net mass of the bioaccessible fraction recovered at each digestion phase (oral, gastric, and intestinal) was determined gravimetrically by weighing the collection tube before and after supernatant recovery. This measured mass was expressed relative to the 200 mg extract input to determine a phase-specific purity factor equal to the proportion of the gross recovered residue mass attributable to the original extract. This gave purity factors of 75.0%, 37.5%, and 36.6% for the oral, gastric, and intestinal phases, respectively (Supplementary Table S5).

The lyophilized residue from each phase was then redissolved in methanol to precipitate residual digestive enzymes and their autolysis products. The precipitate was removed by centrifugation (2500× *g*, 30 min, 4 °C), and only the methanol-soluble supernatant was retained and used for the determination of total phenolic content, total flavonoid content, total flavonol content, and antioxidant activity (Section 2.7 and Section 2.8). Since the purity factor for each sample was already known from the gravimetric determination, it was incorporated directly into the calculation of all results, correcting for the proportion of impurities (carried over digestive components) present in each sample.

### 2.7. Colorimetric Estimation of Polyphenols

The total phenolic content (TPC), total flavonoid content (TFC), and total flavonol content (FOL) were determined using colorimetric assays. The Folin–Ciocalteu reagent was used to measure TPC, and the results were expressed as gallic acid equivalents (µg GAE/mg extract). The aluminum trichloride method was used to quantify TFC and flavonols, and the results were expressed as quercetin equivalents (µg QE/mg extract) and rutin equivalents (µg RE/mg extract), respectively. In all assays, calibration curves were constructed using the corresponding standard compounds and a microplate reader (FLUOstar Omega, BMG Labtech, Ortenberg, Germany) was used [28].

### 2.8. Antioxidant Activity

To evaluate the effect of in vitro gastrointestinal digestion on antioxidant capacity, non-digested samples and lyophilized supernatants from oral, gastric, and intestinal digestion phases (7.81–1000 µg/mL) were dissolved in methanol prior to analysis. The 2,2-diphenyl-1-picrylhydrazyl (DPPH•) radical-scavenging activity was determined by mixing equal volumes of sample dilutions with a methanolic DPPH• stock solution (0.04 mg/mL, initial absorbance adjusted to 0.80 ± 0.02 at 517 nm), incubating for 30 min, and recording the absorbance at 516 nm using a microplate reader. A calibration curve using Trolox was prepared in the same conditions (0.5–10 µg/mL). A control containing methanol instead of the sample was used as a negative control, and the results were corrected against a blank using samples diluted in methanol.

The 2,2′-azino-bis(3-ethylbenzothiazoline-6-sulfonic acid) (ABTS•+) radical was generated by mixing the ABTS solution (7 mM) with 13.24 mg of potassium persulfate for 16 h. The resulting solution was refrigerated and then diluted to reach an absorbance of 0.70 ± 0.02 at 734 nm. ABTS radical cation scavenging capacity was assayed in a 96-well microplate by mixing 18 µL of the sample (7.81–1000 µg/mL) with 182 µL of working ABTS•+ solution, incubating for 6 min, and reading the absorbance at 734 nm. Trolox was used as a reference compound to prepare a calibration curve (19.35–2500 µM).

Trolox equivalent was calculated by selecting a concentration (125 µg/mL) in the linearity range. For DPPH and ABTS, the results were also expressed as IC_50_ (inhibitory concentration), defined as the extract concentration required to neutralize 50% of free radicals; in this case, the lower the value, the greater the antioxidant activity. IC50 was calculated using full concentration–response curves (7.81–1000 µg/mL) (Tables S3 and S4).

The oxygen radical absorbance capacity (ORAC) assay was performed in a 96-well microplate by mixing 40 µL of sample (40 µg/mL) (or potassium phosphate buffer for the blank) with 120 µL of sodium fluorescein (132.5 nM in 10 mM potassium phosphate buffer) and measuring initial fluorescence at an excitation wavelength of 485 nm and an emission wavelength of 520 nm. Peroxyl radical generation was initiated by adding 40 µL of 2,2′-azobis(2-methyl-propionamidine) dihydrochloride (AAPH, 0.3 M in 10 mM potassium phosphate buffer), with fluorescence recorded every 45 s for 1 h to determine the net area under the curve (AUC). The results were quantified against Trolox calibration curves (4–250 µM) and expressed as µM TE/mg extract [24].

### 2.9. UHPLC-DAD-ESI-MS^n^

The identification of individual phenolic compounds was performed using a UHPLC-DAD-ESI-MS^n^ method as described in the work of Righi et al. [27]. The chromatographic analysis was conducted using an Ultimate 3000 instrument from Dionex Co. (Sunnyvale, CA, USA), which was equipped with an Ultimate 3000 Diode Array Detector (DAD) from the same manufacturer. This setup was coupled to a Thermo LTQ XL ion trap mass spectrometer, featuring an ESI source (Thermo Scientific, San Jose, CA, USA). For the separation, a Hypersil Gold C18 column (Thermo Scientific, Waltham, MA, USA) with dimensions of 100 mm length, 2.1 mm inner diameter, and 1.9 µm particle size was employed. The column temperature was maintained at 30 °C throughout the analysis. The elution system operated at a flow rate of 200 µL/min and consisted of two solvents: water/formic acid (99:1, *v*/*v*) as solvent A and acetonitrile/formic acid (99:1, *v*/*v*) as solvent B. The elution program followed these parameters: 5–40% B over 0–15 min, 40–100% B from 15 to 19 min, and then a return to initial conditions, with a total runtime of 34 min. UV detection spanned the wavelength range of 200–500 nm. Simultaneously, the mass spectrometer (Thermo LTQ XL, Thermo Scientific) operated in negative ionization mode. Before injection, samples were dissolved in methanol (10 mg/mL) and filtered using a 0.45 μm syringe filters, and then 10 µL of the filtered solution was injected. Compound identification was accomplished by correlating their retention times, chromatographic behavior and mass spectra with existing literature data.

### 2.10. Statistical Analysis

Statistical analysis was performed using GraphPad Prism software 9.0.0 (121) (GraphPad Software Inc., San Diego, CA, USA). All experiments were conducted in triplicate, and the results were expressed as mean values with standard deviation. To compare the differences between non-digested and digested samples, a one-way analysis of variance (ANOVA) was followed by Tukey’s post hoc test at a significance level of 0.05. IC_50_ was calculated using nonlinear curve fitting (Growth sigmoidal–Boltzmann equation), providing a 95% confidence interval (CI 95%).

## 3. Results and Discussion

### 3.1. Chemical Composition of B. ferulaceum Seeds

The proximate analysis of *Bunium ferulaceum* seeds (BFS) revealed that they were mainly composed of carbohydrates, protein, and ashes, along with a moderate amount of crude lipids (Table 1). Carbohydrates constituted most of the seeds (38.8 ± 2%) with uronic acids, glucose, and mannose as the primary monosaccharide residues. Compared to seven other apiaceous seeds, BFS showed lower carbohydrate and lipid contents, with higher protein and ash contents, but with a comparable energy value [7].

To further investigate the BFS polysaccharides, different fractions were obtained using different solvents. Ethanol extraction was used to remove low-molecular-weight molecules soluble in ethanol, like polyphenols and free sugars. Total sugar concentration in the ethanol-soluble fraction (ESF) was 7.5%. The monosaccharide composition was strongly dominated by mannose (57.2% mol), indicating mannose-rich oligosaccharides or free mannose. Moderate levels of glucose (12.2% mol), galactose (11.0% mol), xylose (10.7% mol), and arabinose (8.2% mol) are present, with trace amounts of rhamnose and fucose.

The analysis of the ethanol-insoluble fraction (EIF) showed that carbohydrates represented 73.1% of the EIF fraction, mainly uronic acids, followed by glucose and mannose (Table 2). The hot-water extraction of the EIF produced a water-soluble fraction (WSF) containing 12.5% carbohydrates, mainly glucose, arabinose, and uronic acids, and a water-insoluble fraction (WIF) with a higher carbohydrate content (40%), rich in glucose, mannose and uronic acids.

Comparatively, coriander seeds [29] and anise seeds [30] exhibited similar glucose and mannose compositions but had higher levels of galactose and lower levels of uronic acids. Black cumin (*Nigella sativa*) seeds showed a similar total carbohydrate content (40%), but the water-soluble fraction contained more neutral sugars (22%) such as arabinose and galactose and higher uronic acids (47%) [31] compared to BFS in the present study. Also, ajwain (*Trachyspermum ammi*) water-soluble polysaccharides were primarily composed of mannose, galactose, and glucose [32].

### 3.2. Fatty Acid Profile

Although seed spices and herbs are not a major source of fatty acids due to their small proportion in the diet, the cumulative nutritional contribution of spices could be significant if utilized more extensively in daily meals. Thus, studying their fatty acid composition can help identify those with health benefits [33]. The fatty acid profile of BFS is shown in Supplementary Materials, Table S1. Petroselinic acid (18:1n-12, cis-Δ6), a unique fatty acid primarily found in apiaceous plants, is challenging to isolate in standard GC method conditions and its peak co-elutes with oleic acid (18:1n-9, cis-Δ9) due to structural similarity [6,7,33]. Accordingly, data in Table S1 are presented as the sum of these two fatty acids.

The fatty acid composition of BFS revealed a rich profile dominated by oleic/petroselinic acid, which constitutes 53.74 g/100 g of the total fatty acids, making them the main components and a significant source of monounsaturated fatty acids (MUFAs), reaching 55.48 g/100 g (Table 3). Petroselinic acid shows antibacterial, antifungal, and anti-inflammatory activities, supporting its use in food and pharmaceutical industries. Its low energy yield suggests potential as a healthier dietary fat [4,6,7,33]. Anise (*Pimpinella anisum*) and caraway (*Carum carvi*) seeds exhibited comparable oleic/petroselinic acid content [6]. In contrast, fennel (*Foeniculum vulgare*) and coriander (*Coriandrum sativum*) seeds contain higher levels of these fatty acids [4,6]. Except for coriander, all other species analyzed presented a lower total unsaturated fatty acid content (≤69%) and a higher total saturated fatty acid content (≥30%) [6]. Cumin (*Cuminum cyminum*) seeds were primarily composed of oleic/petroselinic acids (62%) [4]. All studied apiaceous seed spices are characterized by the predominance of unsaturated fatty acids [6,7,33], a characteristic of all seeds growing in temperate climates. Additionally, BFS contains a substantial amount of polyunsaturated fatty acids (PUFAs), while the saturated fatty acids (SFAs) were relatively low (Table 3).

Considering the elevated risk of cardiovascular diseases (CVDs) and other chronic diseases associated with the intake of saturated fatty acids (SFAs), fats with a polyunsaturated-to-saturated fatty acids (PUFAs/SFAs) ratio lower than 0.45 are generally discouraged in dietary practices [33]. The nutritional quality indices of the fatty acid profile of BFS are shown in Table 3. The PUFA-to-SFA ratio of 2.48 in BFS indicates a more advantageous proportion of unsaturated-to-saturated fats, which is known to contribute positively to cardiovascular health. The combined PUFA-and-MUFA-to-SFA ratio is even more advantageous at 6.83, emphasizing the dominance of heart-healthy unsaturated fats in these seeds. In the study of Saini et al. [33], *Apiaceae* family spices like dill (*Anethum graveolens*), celery (*Apium graveolens*), caraway, coriander and fennel had a PUFA-to-SFA ratio higher than 0.45, while only cumin and parsley (*Petroselinum crispum*) presented a higher PUFA-to-SFA ratio compared to *B. ferulaceum* seeds.

Excessive n-6 polyunsaturated fatty acid (PUFA) consumption elevates the n-6-to-n-3 PUFA ratio, which has been implicated in the pathogenesis of various diseases, including cardiovascular disease, diabetes, depression, immune dysfunction, and neurological disorders [34]. To mitigate these risks, it is recommended to maintain an n-6-to-n-3 PUFA ratio below 4 [34]. The fatty acid composition of BFS resulted in a favorable omega-6 (n-6) to omega-3 (n-3) ratio of 3.86, which supports several health-promoting effects. Parsley presented an adequate n-6-to-n-3 PUFA ratio lower than 4; dill, celery, caraway, coriander, cumin and fennel had very high n-6-to-n-3 PUFA ratios, ranging between 20 and 85 [33]. Importantly, the atherogenic index (IA) and thrombogenic index (IT) of BFS, which are indicators of the potential for promoting atherosclerosis, blood clots, atheroma, and thrombus formation [33], were 0.14 and 0.17, respectively. These low indices with a high hypocholesterolemic-to-hypercholesterolemic fatty acid ratio (h/H) (16.46) suggest a nutritionally advantageous fatty acid profile from a compositional standpoint, consistent with lower theoretical atherogenic and thrombogenic potential [33].

### 3.3. Amino Acid Profile

To determine the amino acid profile of *B. ferulaceum* seeds, proteins were extracted using alkaline extraction at pH = 10, followed by acid precipitation at pH = 4. The resulting protein-rich fraction was neutralized, frozen (−80 °C), and then freeze-dried. The extraction yield of this fraction was 15 ± 2% of the starting seed dry weight, and its protein purity, determined by the Kjeldahl method (N × 6.25), was 67 ± 2%, giving an overall protein recovery rate of 50 ± 8% relative to the whole-seed Kjeldahl protein content. All amino acid results reported in this study (Table S2, Section 3.3) are expressed relative to the protein content of this isolated fraction, and describe the protein-rich fraction specifically, not whole-seed protein.

The amino acid profile of BFS seeds, shown in Table S2, revealed a comprehensive composition of both essential (EAAs) (30.0 ± 2.0%) and non-essential amino acids (NEAAs) (38.0 ± 3.0%). The total amino acid (TAA) content (69.0 ± 5.0%) was comparable to reported values for other plant proteins such as alfalfa sprout protein concentrate (64%) [35]. The profile was dominated by arginine (109.0 ± 47.0 mg/g protein), which is notable for its potential ability to prevent cardiovascular diseases [36]. This was followed by threonine (60.0 ± 6.0 mg/g protein), leucine (57.0 ± 12.0 mg/g protein), and glutamic acid (55.0 ± 5.0 mg/g protein). The high content of glutamic and aspartic acids suggests an acidic character for the BFS protein [37], while glutamic acid specifically is reported to stimulate the immune and central nervous systems [38,39]. Furthermore, the presence of lysine, histidine, and tyrosine in BFS seeds suggests potential antioxidant properties [38,39]. The total essential amino acid (EAA) proportion was comparable to those reported for chia seed protein concentrate at 37% [36], *Moringa oleifera* seeds at 25–27% [40,41], and brewer’s spent grain at 35.5% [42]. Among the EAAs, major constituents include leucine (57.0 ± 12.0 mg/g protein), phenylalanine (52.0 ± 2.0 mg/g protein), and valine (40.0 ± 4.0 mg/g protein). This concentration of branched-chain amino acids (leucine, isoleucine, and valine) is significant as they are essential for muscle building and endurance enhancement [38,39]. Additionally, BFS seeds provide sulfur-containing amino acids, methionine and cystine (21.5 mg/g protein total), which are often rare or limiting in plant proteins [38,43]. While tryptophan was not detected in this profile, a common occurrence in plant protein analysis due to its destruction during acid hydrolysis [35,37], the overall EAA levels suggest that BFS seeds could be a high-quality nutritional source capable of supporting growth [44]. However, the conditions of protein extraction should be optimized to obtain a higher yield and protein recovery rate.

### 3.4. Mineral Composition

With the rising consumption of spices and herbs in everyday diets, understanding their mineral content has gained importance, as these elements play a crucial role in nutrition and overall health [45]. Accordingly, the mineral composition of BFS was determined for four macroelements (K, Ca, Mg, Na) and four microelements (Fe, Mn, Zn, Cu), as shown in Table 4.

*B. ferulaceum* seeds exhibited a rich and well-balanced mineral composition. Macroelements were abundant in the order K > Ca > Mg >Na, with potassium as the most prevalent, supporting myocardial and neuromuscular functions [46,47]. Magnesium content was particularly high, which is a key mineral in enzymatic reactions linked to metabolism and protein synthesis [48]. Overall, these macroelement levels surpassed those reported for several seeds from the Apiaceae family (*Carum carvi*, *Coriandrum sativum*, *Cuminum cyminum*, *Foeniculum vulgare*) [7]. The favorable Na/K (≤2) and Ca/Mg (1.34) ratios further highlight their cardiovascular and mineral bioavailability benefits [47,49]. BFS was exceptionally rich in iron, supporting oxygen transport and immune function [48]. Significant levels of zinc and manganese contribute to DNA synthesis, reproductive health, enzyme activity, and macronutrient metabolism [48]. Copper was also notable, essential for collagen synthesis, iron metabolism, and antioxidant defense [50]. In contrast, other *Apiaceae* seeds contain these microelements mainly at trace levels, with iron remaining their dominant micronutrient [7].

### 3.5. Essential Oil Characterization

The GC-MS analysis of the EOs led to the identification of a total of 22 compounds, representing 79% of the essential oils, and the results are shown in Table 5.

The Clevenger apparatus extraction of *B. ferulaceum* seeds yielded 0.217% of essential oils (EOs), higher than the EO yield of the aerial parts of the same species (0.09%), *Bunium alpinum* (0.1%) [10] and coriander (0.1%) [55]. On the other hand, caraway, aniseed, fennel and cumin presented higher EO yields, ranging from 0.3 to 1.2% [55].

The EOs of *B. ferulaceum* seeds (also referred to as *Bunium incrassatum*) exhibited a chemical composition characterized by sesquiterpene hydrocarbons and oxygenated sesquiterpenes. Sesquiterpene hydrocarbons accounted for a significant portion of the oil’s profile, making up 77.57% of the total composition, and oxygenated sesquiterpenes constitute 4.02% of the total EOs. Among the sesquiterpene hydrocarbons, β-caryophyllene (23.88%) is the major compound, followed by germacrene-B (15.81%) and β-cubebene (14.96%). These compounds are notable for their anti-inflammatory, analgesic, and antimicrobial properties [56]. *Trans*-β-farnesene (12.45%) is another significant component, known for its potential use in pest management due to its role as a natural insect repellent [57]. γ-Elemene (2.14%) and caryophyllene oxide (2.90%), an oxygenated sesquiterpene, further enhance the oil’s bioactivities [58]. Essential oils of the same species from the Oum-El-Buoaghi province (Eastern Algeria) presented a similar composition, but with different proportions for the individual compounds. The proportion of β-caryophyllene (7.2%) *trans*-β-farnesene (8.7) and germacrene-B (5.8%) [17] was low compared to their proportion in *B. ferulaceum* essential oils from the Bordj Bou Arreridj province in the present study, while the proportion of caryophyllene oxide (31%) [17] was very high in *B. alpinum* and *B. incrassatum* compared to the present results. Notably, β-cubebene, one of the main compounds in *B. ferulaceum* essential oils from Bordj Bou Arreridj, was not reported in the same species from the Oum-El-Buoaghi province [17]. The diverse chemical profile of *Bunium ferulaceum* essential oils underscores their potential as a source of bioactive compounds with various nutraceutical and pharmaceutical applications.

### 3.6. Polyphenol Content Under In Vitro Digestion

**Figure 1:**
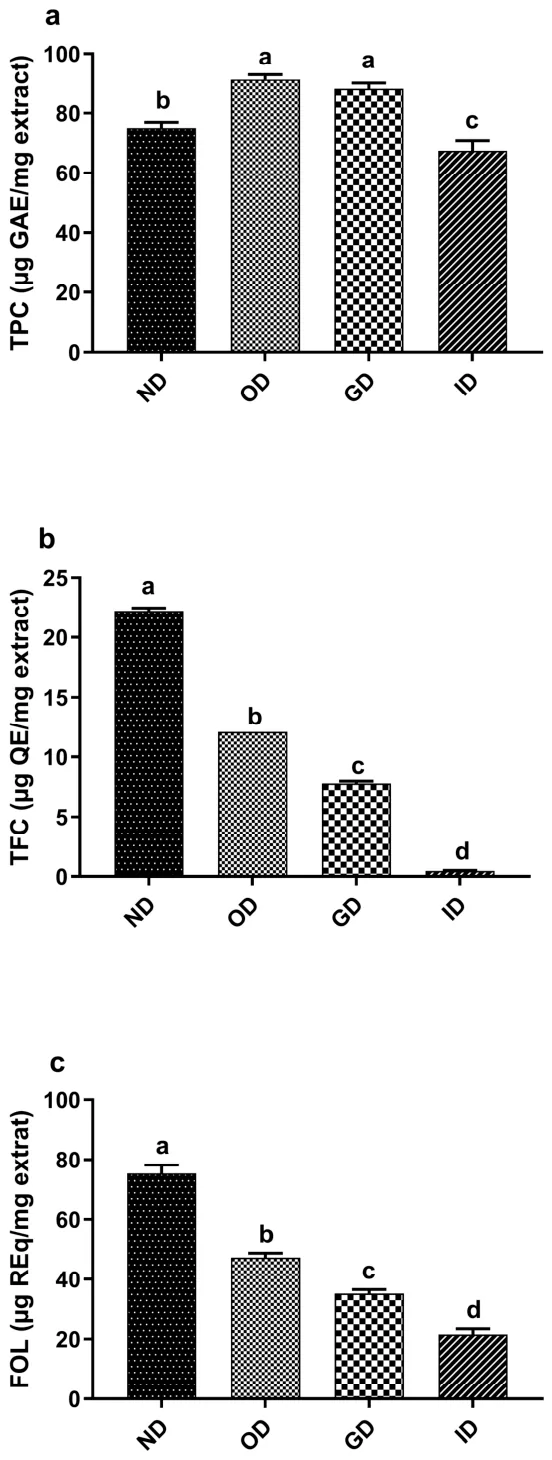
Effect of in vitro simulated gastrointestinal digestion on phenolic content of *Bunium ferulaceum* seeds extract. (**a**): total polyphenol content (TPC); (**b**): total flavonoid content (TFC); (**c**): total flavonol content (FOL). ND: non-digested; OD: oral digestion; GD: gastric digestion; ID: intestinal digestion. Columns with different letters are considered significantly different using one-way analysis of variance (ANOVA), followed by Tukey’s post hoc test at a significance level of 0.05.

The in vitro gastrointestinal digestion of BFS extract significantly impacted its phenolic content, including total polyphenols (TPC), flavonoids (TFC), and flavonols (FOL) (Figure 1). Initially, undigested seeds showed a polyphenol content of 75.08 ± 1.91 µg GAE/mg extract, which significantly increased to 91.18 ± 1.67 µg GAE/mg extract during oral digestion (Figure 1a). On the contrary, a significant decrease in TPC after oral digestion was reported by Sun et al. [59]. Subsequent gastric digestion did not affect the polyphenol content (88.11 ± 1.93 µg GAE/mg extract), with a similar amount compared to the previous oral phase. The increase in total polyphenols after oral digestion and its stability under gastric digestion was possibly due to the release of phenolic compounds bound to other matrix components under the action of pepsin and acidic pH during the gastric phase [60,61,62]. This increase can be attributed to acidic hydrolysis of phenolic glycosides to their aglycons during gastric digestion [63]. Following intestinal digestion, the polyphenol content dropped significantly to 67.44 ± 3.42 µg GAE/mg extract (Figure 1a). The results were in agreement with [62], which reported a loss of almost 50% of TPC after intestinal digestion. This decrease indicates that while initial digestion stages may enhance the availability of polyphenols, the harsh conditions (alkaline pH, digestive enzymes) in the gastrointestinal tract ultimately degrade these compounds [63,64,65] or transform them into less biologically active compounds [66].

The flavonoid (Figure 1b) and flavonol (Figure 1c) contents exhibited a more pronounced decline throughout digestion. The results were in agreement with the reported continued decrease in flavonoids through the gastrointestinal digestion process [61]. Intestinal conditions such as alkaline pH and hydrolytic enzymes may lead to the decrease or even complete disappearance of some compounds, particularly flavonoids, causing their low bioaccessibility [63,65,67]. Despite this, an increase in flavonoid content after gastric digestion has been reported [62].

*B. ferulaceum* seeds from Tlemcen province (Western Algeria) presented comparable total polyphenol content (71–101 µg GAE/mg extract) for different solvents, including aqueous, chloroform and ethyl acetate [19]. The same study showed that methanol and petroleum ether extracts contained higher total polyphenol content. In our study, even after complete in vitro gastrointestinal digestion, BFS presented higher total polyphenol content compared to aqueous and ethanolic seeds extracts of coriander, adjwain, fennel, caraway, dill, and anise [8]. On the other hand, methanolic extracts of wooly cumin (10 mg GAE/g d.w), parsley (11 mg GAE/g d.w), and anise (11 mg GAE/g d.w) [8] showed total polyphenol content comparable to that of BFS, 11.25 mg GAE/g dry seed weight. However, methanolic extracts from fennel (28 mg GAE/g d.w), cumin (21 mg GAE/g d.w) and coriander (18 mg GAE/g d.w) [7] presented higher total polyphenols.

The flavonoid content in acetone/water extracts of caraway, coriander cumin and mystical cumin [5] was also lower than it was in the undigested BFS and comparable to the flavonoid content in the intestinal digestion of BFS. These findings pointed out the richness of BFS compared to other apiaceous seeds used as spices.

### 3.7. Antioxidant Activity During In Vitro Digestion

**Figure 2:**
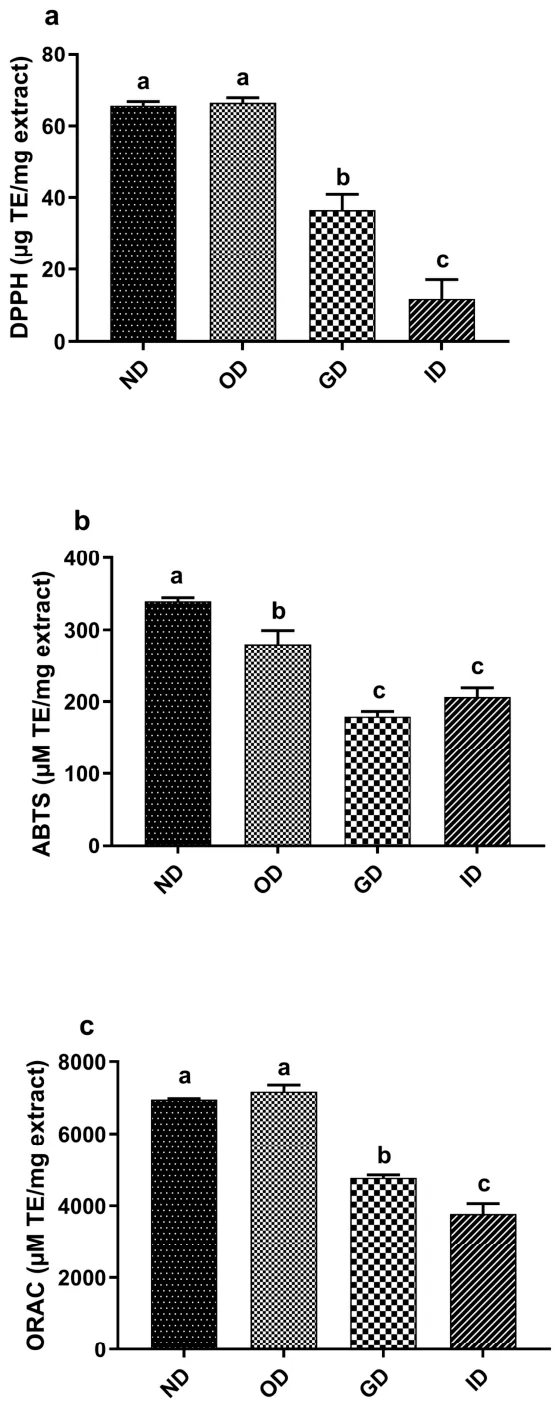
Effect of in vitro simulated gastrointestinal digestion on antioxidant activity of *Bunium ferulaceum* seeds extract. (**a**): DPPH radical inhibition; (**b**): ABTS radical inhibition; (**c**): ORAC assay. ND: non-digested sample; OD: oral digestion; GD: gastric digestion; ID: intestinal digestion. Columns with different letters are considered significantly different using one-way analysis of variance (ANOVA), followed by Tukey’s post hoc test at a significance level of 0.05.

The results obtained from the different assays used to measure the antioxidant activity during the digestion process are shown in Figure 2. For the DPPH assay, undigested seeds exhibited an antioxidant activity of 65.65 ± 1.10 μg TE/mg extract (Figure 2a), which remained stable after oral digestion. However, a substantial decrease of 44% occurred during gastric digestion, lowering the antioxidant activity to 36.53 ± 4.38 μg TE/mg extract. This decline was further pronounced during intestinal digestion, with antioxidant activity dropping by 67.78% compared to the gastric phase and by 82% compared to the undigested sample. The same trend was observed for the ORAC assay (Figure 2c)**,** which demonstrated stable activity after oral digestion but then showed a marked decrease of 33.42% during gastric digestion compared to the previous phase and further decreased by 20.96% during post-intestinal digestion compared to the gastric phase. On the other hand, the ABTS assay (Figure 2b) showed a significant reduction in antioxidant activity from the beginning of the digestion, decreasing by 17.41% from 339.42 ± 4.99 μM TE/mg in undigested seeds to 280.33 ± 18.94 μM TE/mg after oral digestion. This was followed by a further 36.00% decrease during gastric digestion in comparison to the oral phase, and then it remained stable at 206.39 ± 12.68 μM TE/mg in the intestinal phase. We previously reported a similar pattern for the ethyl acetate extract of *Ranunculus macrophyllus* aerial parts [24]. An increase in the antioxidant activity of *Sorghum bicolor* during the first stage of digestion, followed by a gradual decrease during the following stages, was also reported [68]. In general, antioxidant activity decreases during gastrointestinal digestion [69,70], although there are some studies [60] reporting increased antioxidant activity due to heat treatments of the sample, which can lead to the release of polyphenols from the food matrix.

The decrease in antioxidant activity during gastric and intestinal digestion aligns with the degradation of flavonoids and flavonols (Figure 1b,c). This suggests that the antioxidant potential of BFS is closely linked to its flavonoid content, which undergoes significant reduction under the harsh conditions of gastrointestinal digestion, leading to diminished antioxidant capacity. Moreover, the sensitivity of different antioxidant assays (DPPH, ABTS, ORAC, FRAP) to various antioxidant types means that an increase in TPC may not directly correspond to an increase in activity detected by these methods [71]. Finally, interactions between polyphenols and other digestive components, such as proteins and lipids, can lead to the formation of complexes or the polymerization of polyphenols, further reducing their antioxidant efficacy despite an increase in their measured content [61,71,72]. These factors may collectively explain the disparity between the trends in polyphenol content and antioxidant activity.

To compare our results to other apiaceous seeds, DPPH and ABTS IC_50_ values (inhibitory concentration that neutralizes 50% of free radicals; in this case, the lower the value, the greater the antioxidant activity) were also calculated for the undigested and digested samples. Undigested BFS showed the highest radical-scavenging ability (IC_50_ = 73 µg/mL, 95% CI: 67.78–77.97 µg/mL) compared to wooly cumin, caraway, coriander, cumin, fennel, parsley and anise, which showed a DPPH radical-scavenging ability ranging from 118 to 369 µg/mL [7]. Even after full gastrointestinal digestion, the DPPH radical-scavenging ability of BFS (IC_50_ = 173 µg/mL, 95% CI: 150–195.3 µg/mL) was still higher compared to all previous species except fennel (IC_50_ = 118 µg/mL). The ABTS radical-scavenging ability of undigested BFS (IC_50_ = 339 µg/mL, 95% CI: 320.10–418.16 µg/mL) was higher compared to wooly cumin, parsley and anise (IC_50_: 392–429 µg/mL), comparable to that of caraway (IC_50_ = 329 µg/mL), and lower compared to coriander, cumin, and fennel (IC_50_ = 134–294 µg/mL) [7]. The differences observed can be linked to the different polyphenols and flavonoid content among the different species.

While gastrointestinal digestion does impact the antioxidant activity of BFS, its overall antioxidant capacity remains notably higher than that of many other apiaceous seeds even after digestion. This suggests that *B. ferulaceum* could be a valuable source of natural antioxidants with potential applications in various fields, including the food, pharmaceutical, and cosmetic industries.

### 3.8. UHPLC-DAD-ESI-MS^n^ Analysis

**Figure 3:**
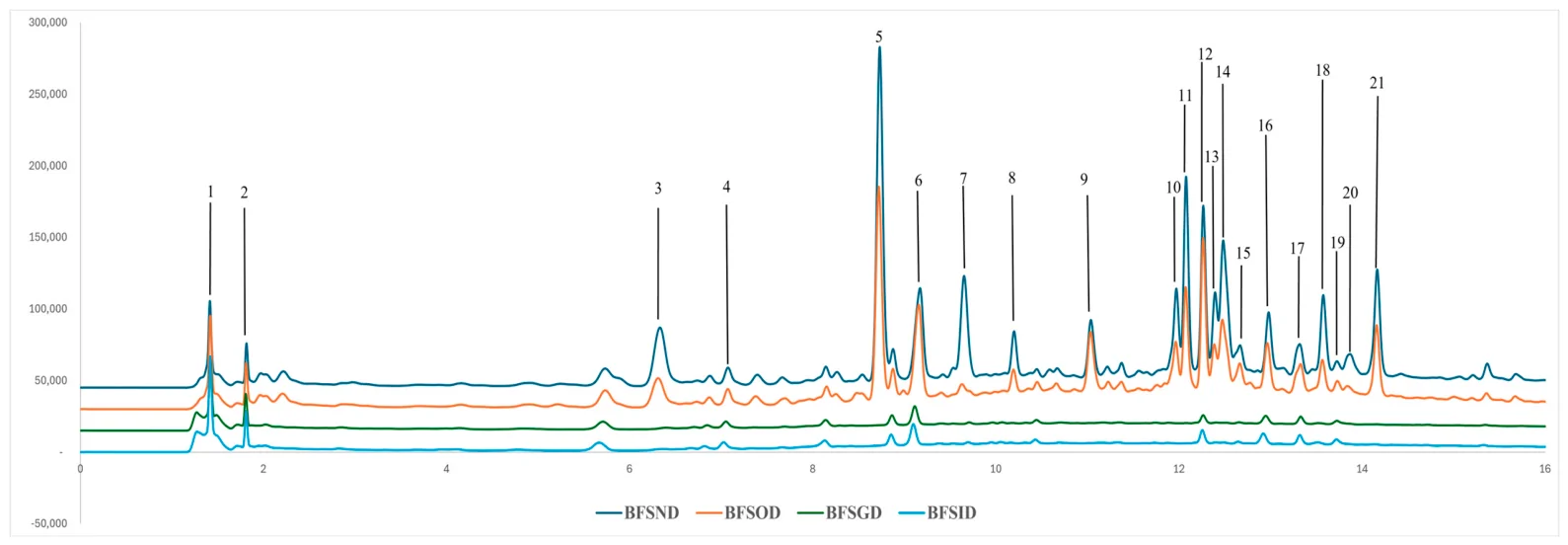
Chromatograms of *Bunium ferulaceum* seeds extract at 280 nm. BFS ND: *Bunium ferulaceum* seeds non-digested extract; BFS OD: *Bunium ferulaceum* seeds extract oral digestion; BFS GD: *Bunium ferulaceum* seeds extract gastric digestion; BFS ID: *Bunium ferulaceum* seeds extract intestinal digestion. Peak labels refer to the peak numbers (identified compounds) in Table 6.

The phytochemical analysis of BFS extract before and after the different steps of digestion process (Figure 3) revealed a diverse profile of polyphenolic compounds, predominantly composed of hydroxycinnamic acids and flavonols (Table 6). The identification of the main peaks in the BFS extract was carried out through UHPLC-DAD-ESI-MS^n^, using retention times, UV behavior, molecular ions, and characteristic fragmentation patterns to determine their structures in comparison to available literature.

Overall, the hydroxycinnamic acids comprised mainly caffeic acid derivatives. Peaks 3, 5 and 8 exhibited an [M-H]^−^ ion at *m*/*z* 353, indicating caffeoylquinic acid derivatives. Peak 3 was tentatively assigned as 3-*O*-caffeoylquinic acid, showing UV-λ_max_ at 295 and 323 nm, alongside a strong *m*/*z* 179 and *m*/*z* 191 [73,74]. Peak 5 was tentatively assigned as 4-*O*-caffeoylquinic acid, exhibiting UV-λ_max_ at 291 and 325 nm and a characteristic base peak at *m*/*z* 173 [73,75]. Peak 8 was tentatively assigned as 5-*O*-caffeoylquinic acid, displaying UV-λ_max_ at 290 and 323 nm and a major MS^2^ base peak at *m*/*z* 191, with a minor peak at *m*/*z* 179 [73,75]. Peak 4 showed UV-λ_max_ at 291 and 316 nm, with a precursor ion at *m*/*z* 193 and MS^2^ fragments at *m*/*z* 134, 149, and 178, possibly corresponding to ferulic acid [76]. Peak 6, tentatively corresponding to feruloylquinic acid, showed UV-λ_max_ at 290 and 325 nm, an [M-H]^−^ ion at *m*/*z* 367 and fragments at *m*/*z* 193 and 173, indicative of ferulic acid cleavage [77]. Peak 9 exhibited UV-λ_max_ at 290 and 325 nm, a UV spectrum similar to that of caffeic acid derivatives. Moreover, Peaks 19, 20, and 21 were tentatively assigned to di-caffeoylquinic acid isomers for exhibiting an [M-H]^−^ ion at *m*/*z* 515, with UV-λ_max_ at 290 and 325 nm (peaks 20 and 21) and fragments at *m*/*z* 353, indicative of sequential loss of caffeoyl [78].

Peaks 10–18 were assigned to flavonoids and their derivatives as follows: Peak 10 was tentatively identified as quercetin-*O*-hexosyl-pentoside, exhibiting UV-λ_max_ at 354 nm, an [M-H]^−^ ion at *m*/*z* 595 and a characteristic fragment 463 due to the loss of pentosyl and 301 (quercetin aglycon) due to the loss of pentosyl and hexosyl moieties [79]. Peaks 11–17 were tentatively identified as quercetin derivatives, exhibiting a characteristic fragment of quercetin aglycon (301) and UV-λ_max_ around 350–360 nm [80]. Peak 13 had a UV-λ_max_ at 353 nm, [M-H]^−^ at *m*/*z* 593 → 285, with the fragment at *m*/*z* 285 corresponding to kaempferol aglycon resulting from the loss of the rutinosyl moiety; the peak was tentatively identified as kaempferol-*O*-rutinoside [81,82]. Peak 15, presenting UV-λ_max_ at 351 nm, a parent ion [M-H]^−^ at *m*/*z* 477 with an MS^2^ main fragment at *m*/*z* 301 (M-176, corresponding to the loss of the glucuronide moiety), was tentatively identified as quercetin-*O*-hexuronide; the UV-λ_max_ at 351 nm further corroborates the quercetin (flavonol) skeleton [82,83]. Peak 17 was tentatively assigned to quercetin-*O*-acetylhexoside, with its UV-λ_max_ at 330 nm, [M-H]^−^ at *m*/*z* 505 and the loss of 204 Da (acetylhexoside) to yield quercetin (*m*/*z* 301) [84]. Peak 18, tentatively identified as isorhamnetin-*O*-hexoside, showed UV-λ_max_ at 290 and 327 nm, an [M-H]^−^ at 477 with a characteristic loss of 162 Da, resulting in a fragment at *m*/*z* 315, corresponding to isorhamnetin [85].

Table 6 reports qualitative detection outcomes (presence or absence) for each identified compound under the stated UHPLC-DAD-ESI-MSn analytical conditions. From the initial 21 peaks identified in the seed extract before digestion, only 13 peaks remained detectable at final step of digestion.

Among the compounds identified in BFS, citric acid (Peak 1) and malic acid (Peak 2) remained detectable throughout all stages of in vitro digestion. These organic acids were consistently detected in the non-digested, oral, gastric, and intestinal phases, which is in line with the observations of Igual et al. [86] and Nekvapil et al. [87], who reported high bioaccessibility of organic acids in beetroot and matcha tea, respectively. The stability of organic acids could be attributed to their simple carboxyl structures that are resilient to gastrointestinal pH shifts and enzymatic degradation, with certain acids even being continuously released via the breakdown of complex precursor molecules [86,87].

Similarly, quercetin-O-rutinoside (Peak 12) and ferulic acid derivatives (Peaks 4 and 6) remained detectable through the later digestion phases and may be among the phenolic compounds contributing to the residual antioxidant activity detected in later digestion stages, particularly in the ABTS and ORAC assays (Figure 2b,c). Such a pattern is consistent with other reports showing that rutin and other phenolic acid derivatives may persist, or even increase in detectability, during digestion depending on the food matrix [62,88]. The reduced detectability of some of these compounds during the intestinal phase coincides temporally with the decrease in total polyphenol content (TPC) observed in this phase (Figure 1a), suggesting that hydroxycinnamates may be among the phenolic classes contributing to this decline; confirming this would require quantitative data not available in the present study. The non-detection of certain hydroxycinnamic derivatives (e.g., 5-O-caffeoylquinic acid, other hydroxycinnamic acids) after intestinal digestion is consistent with the possible degradation or structural transformation into less active forms under alkaline conditions since specific degradation products were not identified in this study; a broadly similar pattern has been reported elsewhere [62,65].

Flavonoid glycosides such as quercetin-O-hexoside (Peak 14), quercetin-O-hexuronide (Peak 15), and kaempferol derivatives (Peak 13) were no longer detected in the intestinal-phase samples. This reduced detectability coincides with the declines recorded in TFC and FOL values during gastric and intestinal digestion (Figure 1b,c), consistent with the behavior commonly reported for flavonoids under acidic and alkaline digestive stress [67,68]. Comparable variability in flavonoid presence has been reported elsewhere, where rutin and quercetin losses ranged from minor to complete degradation depending on the matrix and digestion conditions [68]. However, our analytical conditions themselves do not quantify the magnitude of loss.

The changes in phenolic content help account for the complex antioxidant response observed. Although a moderate increase in phenolics during oral and gastric digestion increased or maintained TPC levels (Figure 1a), this did not translate proportionally into higher antioxidant activity. For example, while DPPH and ORAC responses remained relatively stable during oral digestion, all three assays (DPPH, ABTS, ORAC) revealed progressive, and substantial, losses of antioxidant capacity during the gastric and, most markedly, the intestinal phases (Figure 2).

Our findings indicate that the antioxidant potential of BFS extract was highly dependent on its flavonoid fraction, which shows both a substantial quantitative decline (TFC, Figure 1c) and reduced detectability of several individual glycosides during digestion. Initial digestive phases may enhance the release and detectability of phenolic compounds from plant extracts. However, once released, phenolic compounds are more exposed to the harsh conditions of the gastrointestinal tract, which ultimately can limit their functional bioaccessibility and antioxidant activity [89]. Recoveries can range from 20 to 70% for anthocyanins, with losses of 26–43% reported for various polyphenol classes in other extracts [90]. In contrast, within a food matrix, the behavior of polyphenols depends on matrix composition. Dietary fiber can physically trap polyphenols, restricting enzyme access and reducing their immediate release in the upper gastrointestinal tract. Proteins may form complexes with polyphenols that reduce initial bioaccessibility but simultaneously protect them from oxidative degradation during transit, potentially enhancing their stability [90,91]. While some phenolic groups remain detectable after simulated gastrointestinal digestion, their reduced concentration and/or altered structure appear to be associated with a marked decline in antioxidant activity.

## 4. Conclusions

*Bunium ferulaceum* seeds represent a functionally rich botanical matrix with a distinctive compositional profile. The seeds were characterized by a substantial carbohydrate fraction consisting primarily of ethanol-insoluble polysaccharides enriched in uronic acids. The lipid profile featured a high proportion of monounsaturated fatty acids. The protein-rich fraction contained all 20 amino acids, with arginine, threonine and leucine as the predominant amino acids. Additionally, the seeds were found to be a significant source of essential minerals, particularly potassium, calcium, and iron. The volatile composition was dominated by sesquiterpenes, with β-caryophyllene and germacrene-B as major constituents. Phenolic antioxidants, such as caffeoylquinic acid derivatives and quercetin derivatives, were detected in undigested seeds. However, several flavonoid glycosides were no longer detected, particularly during the simulated intestinal phase. Nevertheless, the seeds maintained significant post-digestive antioxidant activity. Beyond their compositional interest, these findings point to concrete avenues for valorizing BFS as a food ingredient. The protein-rich and polysaccharide-rich fractions could be incorporated into bakery or snack formulations as functional additives, while the essential oil offers potential as a natural flavoring or antimicrobial agent. Given that several flavonoid glycosides were no longer detected during the intestinal phase, encapsulation strategies (e.g., microencapsulation or incorporation into a protective food matrix) need investigation to maintain their presence throughout gastrointestinal transit.

## Figures and Tables

**Table 1:** Proximate composition of *Bunium ferulaceum* seeds.

*Bunium ferulaceum* Seeds	
Proteins (g/100 g d.w.b)	19.03 ± 1.17
Lipids (g/100 g d.w.b)	3.62 ± 0.17
Ash (g/100 g d.w.b)	8.87 ± 0.12
Carbohydrates (g/100 g d.w.b)	38.80 ± 2.8
Monosaccharides (% mol)	
Fuc	0.9
Ara	9.6
Xyl	9.2
Man	16.4
Gal	4.9
Glc	26.9
UA	32.1
Energy (kcal/100 g d.w.b)	382.6 ± 1.1

Fuc: Fucose; Ara: Arabinose; Xyl: Xylose; Man: Mannose; Gal: Galactose; Glc: Glucose; UA: Uronic acid. d.w.b: Dry weight basis.

**Table 2:** Sugar composition of *Bunium ferulaceum* seeds and their different fractions.

Sample		Monosaccharide Composition (%mol)								Total Sugars (mg/g)
	Dry Yield (%)	Rha	Fuc	Ara	Xyl	Man	Gal	Glc	UA	Average
ESF	17	0.2	0.6	8.2	10.7	57.2	11.0	12.2	/	75 ± 10
EIF	81	0.0	0.4	5.7	6.6	7.8	1.9	12.3	65.0	731 ± 36
WSF	18	0.00	2.5	14.5	3.8	11.1	11.4	21.3	36.0	125 ± 12
WIF	79	0.01	0.9	13.6	1.4	21.0	4.0	29.0	30.0	400 ± 50

ESF: Ethanol-soluble fraction; EIF: Ethanol-insoluble fraction; WIF: Water-insoluble fraction; WSF: Water-soluble fraction; Rha: Rhamnose; Fuc: Fucose; Ara: Arabinose; Xyl: Xylose; Man: Mannose; Gal: Galactose; Glc: Glucose; UA: Uronic acid. mg/g refers to mg/g of each isolated fraction. ESF and EIF yields are calculated in relation to initial raw material; WSF and WIF yields are calculated in relation to EIF.

**Table 3:** Fatty acids fractions and nutritional quality indices of fatty acids from *B. ferulaceum* seeds.

Quality Indices	
SFAs	12.77 ± 0.09
MUFAs	55.48 ± 0.07
PUFAs	31.75 ± 0.97
N-3	6.53 ± 0.12
N-6	25.22 ± 1.05
N6/N3	3.86 ± 0.21
PUFA/SFAs	2.48 ± 0.05
(PUFA + MUFA)/SFAs	6.83 ± 0.03
Trans FAs	nd
AI	0.14 ± 0.00
TI	0.17 ± 0.00
h/H	16.46 ± 0.00

SFAs: Saturated fatty acids; MUFAs: Monounsaturated fatty acids; PUFAs: Polyunsaturated fatty acids; N-3: Omega 3 (ω3) fatty acids; N-6: Omega 6 (ω6) fatty acids; Trans FAs: Trans Fatty Acids; AI: Atherogenic index; TI: Thrombogenic index; h: Hypocholesterolemic fatty acids; H: Hypercholesterolemic fatty acids; nd: Not detected.

**Table 4:** Macro- and microelements found in *Bunium ferulaceum* seeds.

Mineral Elements (mg/100 g d.w.)	
Potassium	1560 ± 180
Calcium	562 ± 14
Sodium	316 ± 7
Magnesium	420 ± 60
Iron	18.2 ± 2.0
Zinc	5.6 ± 0.6
Manganese	3.0 ± 0.1
Copper	1.5 ± 0.1

**Table 5:** GC–MS characterization of the essential oils extracted from *Bunium ferulaceum* seeds.

No.	Compounds	RI_LIT_	RI_E_	Content (mg/g)	Relative Composition (%)
	Sesquiterpene hydrocarbons				
1	α-cubebene	1366 ^d^	1370	1.33 ± 0.74	0.21 ± 0.00
2	α-copaene	1414 ^a^	1419	3.57 ± 1.00	0.31 ± 0.02
3	Germacrene-D	1481 ^e^	1498	1.20 ± 0.57	0.11 ± 0.03
4	α-calacorene	1542 ^e^	1541	1.55 ± 0.72	0.12 ± 0.00
6	β-caryophyllene	1566 ^a^	1574	246.27 ± 49.34	23.88 ± 0.81
7	ϒ-elemene	1625 ^b^	1644	22.91 ± 1.52	2.14 ± 0.55
8	α- humulene	1658 ^a^	1662	17.09 ± 1.90	1.69 ± 0.19
9	α-morphene	/	1694	7.33 ±0.00	0.75 ± 0.14
10	Trans-β-Farnesene	1695 ^c^	1703	114.97 ± 5.02	12.45 ± 1.79
11	β-cubebene	1612 ^b^	1713	137.76 ± 31.64	14.96 ± 0.56
12	α-amorphene	1708 ^f^	1725	1.22 ± 0.08	0.23 ± 0.15
13	Bicyclogermacrene	1500 ^e^	1737	11.15 ± 2.03	0.27 ± 0.18
14	ϒ-cadinene	1742 ^c^	1766	5.90 ± 3.45	0.42 ± 0.03
15	δ-cadinene	1759 ^b^	1771	8.66 ± 0.46	0.87 ± 0.13
16	β-sesquiphellandrene	/	1790	3.46 ±0.00	0.39 ± 0.02
17	Germacrene-B	1821 ^a^	1824	164.08 ± 25.05	15.81 ± 1.59
18	Aromadendrene	/	1932	2.66 ± 0.00	0.26 ± 0.07
	Oxygenated sesquiterpenes				
5	Cascarilladiene	/	1556	3.60 ± 1.04	0.29 ± 0.00
19	Caryophyllene oxide	1934 ^a^	1937	28.64 ± 1.82	2.90 ± 0.38
20	salvial-4(14)-en-1-one	/	1955	1.64 ± 0.00	0.19 ± 0.00
21	o-Menth-8-ene	/	1973	1.32 ± 0.00	0.16 ± 0.01
22	Spathulenol	2035 ^a^	2037	2.77 ± 0.26	0.32 ± 0.02
Total				789.02 ± 107.53	77.57 ±6.23

[27] ^a^; [51] ^b^; [52] ^c,d^; [53] ^e^, [54] ^f^. RI_LIT_: Literature retention index; RI_E_: Experimental retention index.

**Table 6:** Qualitative analysis of the different samples from *Bunium ferulaceum* non-digested and digested seeds extract using UHPLC-DAD-ESI-MS^n^.

Peak	Rt (min)	λ_max_	[MH]^−^	ESI-MS/MS Product Ions	Probable Compound	Samples			
						ND	OD	GD	ID
1	1.4	210	191	111, 173, 85	Citric acid	+	+	+	+
2	1.8	195	133	115, 105	Malic acid	+	+	+	+
3	6.3	295, 323	353	191, 179, 173	3-*O*-Caffeoylquinic acid	+	+	-	-
4	7.1	291, 316	193	134, 149, 178	Ferulic acid	+	+	+	+
5	8.7	291, 325	353	173, 179	4-*O*-Caffeoylquinic acid	+	+	+	+
6	9.2	290, 325	367	193	Feruloylquinic acid	+	+	+	+
7	9.7	290, 323	595	300, 509	Unknown	+	+	-	-
8	10.2	290, 323	353	191, 179, 161	5-*O*-Caffeoylquinic acid	+	+	-	-
9	11.0	290, 325	507	-	Caffeic acid derivative	+	+	-	-
10	12.0	354	595	300, 301, 463, 475	Quercetin-*O*-hexosyl-pentoside	+	+	-	-
11	12.1	354	609	301, 300	Quercetin-*O*-rutinoside (isom 1)	+	+	-	-
12	12.3	354	609	301, 300	Quercetin-*O*-rutinoside (isom 2)	+	+	+	+
13	12.4	353	593	285	Kaempferol-*O*-rutinoside	+	+	-	-
14	12.5	353	463	301, 300	Quercetin-*O*-hexoside	+	+	-	-
15	12.7	351	477	301	Quercetin-*O*-hexuronide	+	+	-	-
16	13.0	341	505	301, 463, 300, 487	Quercetin-*O*-acetylhexoside	+	+	+	+
17	13.3	330	505 651 515	301, 463 609, 315, 301, 463 353, 335, 173	Quercetin-*O*-acetylhexoside Quercetin-*O*-acetyl- pentosylhexoside Di-*O*-caffeoylquinic acid (isom 1)	+	+	+	+
18	13.6	290, 327	477 515	314, 301, 315, 357, 433 353, 335	Isorhamnetin-O-hexoside Di-*O*-caffeoylquinic acid (isom 2)	+	+	-	-
19	13.7	-	515	353, 335	Di-*O*-caffeoylquinic acid (isom 3)	+	+	+	+
20	13.9	290, 325	515	353, 179	Di-*O*-caffeoylquinic acid (isom 4)	+	+	-	-
21	14.2	290, 325	515	353, 299, 179	Di-*O*-caffeoylquinic acid (isom 5)	+	+	-	-

+, present; - Not detected; Rt: Retention time; ND: Non-digested extract; OD: Extract oral digestion; GD: Extract gastric digestion; ID: Extract intestinal digestion; isom: isomer.

## Data Availability

The raw data supporting the conclusions of this article will be made available by the authors on request.

## Supplementary Materials

The following supporting information can be downloaded at: https://www.mdpi.com/article/10.3390/foods15193552/s1, Table S1: Fatty acid profile (g/100 g fatty acids) of *Bunium ferulaceum* seeds; Table S2: Amino acid profile of protein rich fraction from *Bunium ferulaceum* seeds; Table S3: DPPH concentration response data used to calculate IC_50_; Table S4: ABTS concentration response data used to calculate IC_50_; Table S5: Post-digest dry mass recovery (supernatant); Figure S1: a. Representative MRM (Multiple Reaction Monitoring) chromatograms (Total amino acid profile); b. Glutamic acid; c. glutamine; d. Aspartic acid: e. asparagine; Figure S2: Representative GC-FID chromatogram of fatty acid methyl esters (FAMEs) from the lipidic fraction of *Bunium ferulaceum* seeds.
