# Supplementary material for: Nutritional Composition, Essential Oil Profiling, and Post-Digestion Phytochemical and Antioxidant Properties of Fennel Pignut (Bunium ferulaceum) Seeds
Source: Foods. 2026 Oct 6;15(19):3552. doi: 10.3390/foods15193552 (PMC13664972; doi:10.3390/foods15193552)

Supplementary Materials for:

Title: Nutritional Composition, Essential Oil Profiling, and Post-Digestion phytochemical and Antioxidant properties of Fennel Pignut (*Bunium ferulaceum*) Seeds

A. Deghima, N. Righi, E. Coelho, M. I. Arranca, S. S. Ferreira, F. Baali, S. M. Cardoso, D. Ansorena, I. Astiasarán, M. A. Coimbra

Table S1: Fatty acid profile (g/100 g fatty acids) of *Bunium ferulaceum* seeds

Fatty acid	Rt (min)	mean ± SD
Caprylic	5.054	0.15 ± 0.02
Capric	5.519	0.15 ± 0.04
Lauric	6.091	0.08 ± 0.01
Myristic	7.241	0.45 ± 0.01
Palmitic	9.26	8.03 ± 0.08
Stearic	12.19	1.90 ± 0.02
Oleic/ Petroselinic	13.11	53.74 ± 0.01
Vaccenic	13.22	1.75 ±0.07
Linoleic	14.403	16.72 ± 0.03
Arachidic	14.984	0.97 ± 0.01
α-linolenic	16.037	5.51 ± 0.05
Eicosadienoic (n-6)	17.469	8.44 ± 1.06
Behenic	17.786	0.99 ± 0.01
EPA	20.8	1.00 ± 0.09

Table S2: Amino acid profile of protein rich fraction from *Bunium ferulaceum* seeds

**Amino acids**	g/100 g proteins	mg/g protein
**Essential Amino acids**		
Lysine	4. 0±1.5	37.0±15.0
Histidine	2.0±0.1	19.0±1.0
Threonine	6.0±0.6	60.0±6.0
Valine	4.0±0.4	40.0±4.0
Methionine	2.0±0.2	19.0±2.0
Phenylalanine	5.0±0.1	52.0±2.0
Leucine	6.0±1.0	57.0±12.0
Isoleucine	2.03±0.01	20.30±0.08
Tryptophan	nd	nd
**Total essential A.A**	30.0±2.0	305.0±17.0
**Non-essential amino acids**		
Glycine	3.0±1.0	33.0±10.0
Arginine	11.0±5.0	109.0±47.0
Serine	5.0±1.0	52.0±14.0
Aspartic Acid/Asparagine	4.0±0.4	38.0±5.0
Glutamic Acid/Glutamine	5.0±0.4	55.0±5.0
Alanine	3.0±1.0	34.0±11.0
Proline	3.0±0.1	32.0±1.0
Cystine	0.25±0.04	2.5±0.4
Tyrosine	3.0±0.1	30.0±2.0
**Total NEAA**	38.0±3.0	384.0±30.0
**Total A.A**	69.0±5.0	690.0±48.0

nd: Not detected

Table S3: DPPH concentration response data used to calculate IC_{50}

Concentration-Response table | Concentration-Response Graph

ND Concetrations (µg/ml)	Average inhibition (%)
7.81	7.60
15.63	17.86
31.25	20.60
62.50	40.41
125.00	83.11
250.00	91.95
500.00	90.01
1000.00	87.98

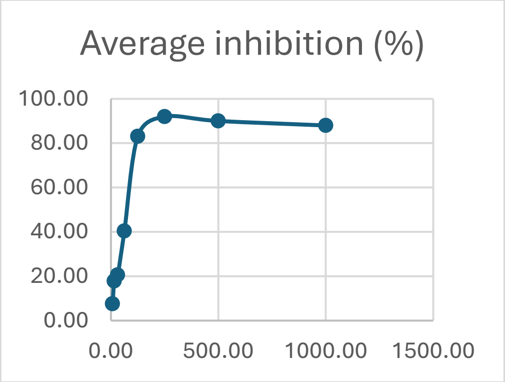

OD Concetrations (µg/ml)	Average inhibition (%)
7.8125	7.08
15.625	10.75
31.25	19.80
62.5	42.83
125	83.33
250	89.70
500	79.03
1000	81.09

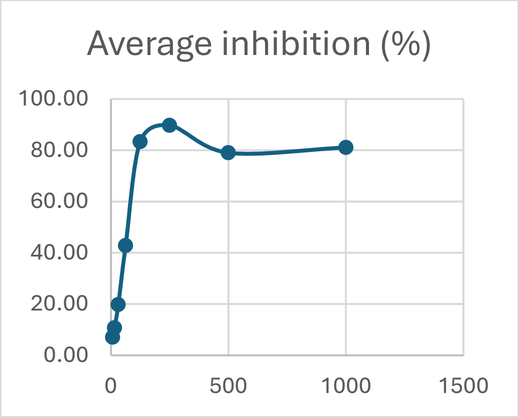

GD Concetrations (µg/ml)	Average inhibition (%)
7.8125	10.59
15.625	10.24
31.25	8.68
62.5	14.41
125	24.48
250	58.16
500	68.84
1000	74.13

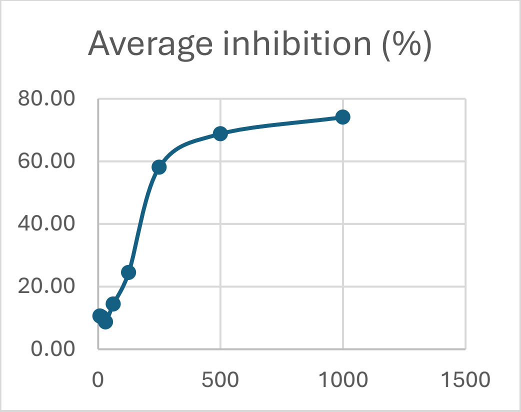

ID Concetrations (µg/ml)	Average inhibition (%)
7.8125	6.34
15.625	5.63
31.25	2.99
62.5	4.84
125	11.88
250	39.35
500	60.74
1000	71.57

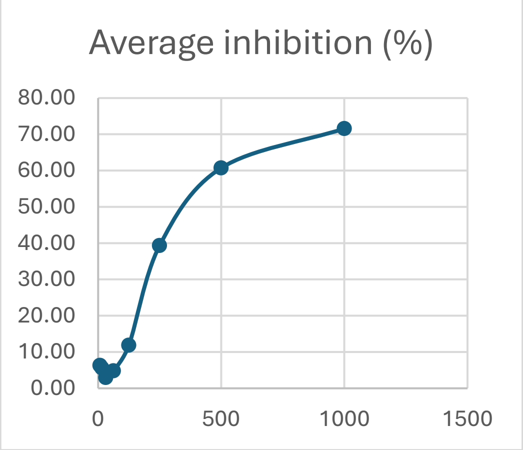

Table S4: ABTS concentration response data used to calculate IC_{50}

Concentration-Response table	Concentration-Response Graph

**ND Concentration (µg/ml)**	Average Inhibition (%)
7.82	1.24
15.63	3.27
31.25	5.91
62.50	11.41
125.00	18.93
250.00	32.83
500.00	60.37
1000.00	83.97

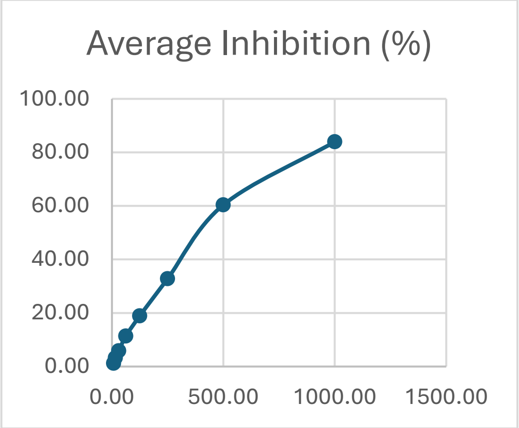

**OD Concentration (µg/ml)**	Average Inhibition (%)
7.82	1.61
15.63	4.30
31.25	8.45
62.50	8.66
125.00	15.61
250.00	30.08
500.00	51.19
1000.00	89.42

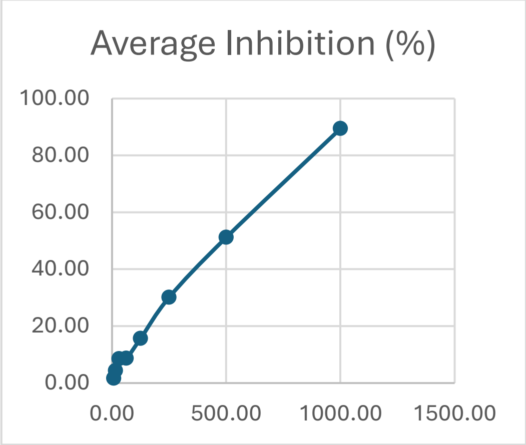

**GD Concentration (µg/ml)**	Average Inhibition (%)
7.82	1.04
15.63	1.19
31.25	2.39
62.50	2.18
125.00	7.42
250.00	11.88
500.00	22.98
1000.00	68.00

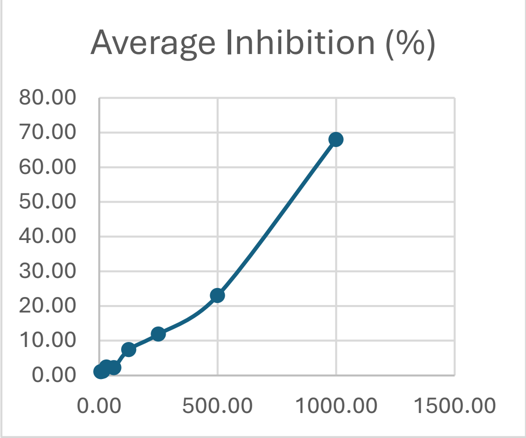

**ID Concentration (µg/ml)**	Average Inhibition (%)
7.815	0.41
15.625	1.14
31.25	1.82
62.5	4.77
125	7.94
250	14.99
500	30.65
1000	61.93

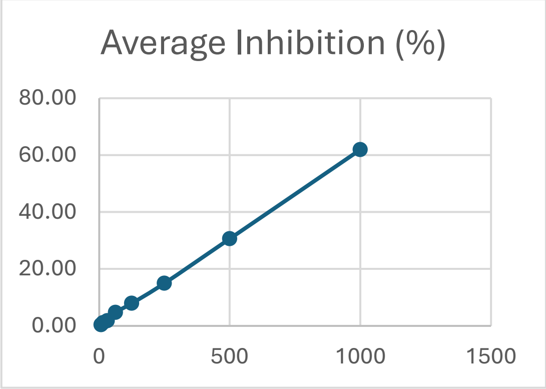

Table S5: Post-digest dry mass recovery (supernatant)

Oral Digestion	Falcon (F)	F + Supernatant	NET	Supernatant (mg) (Dry mass)	Purity Factor	Avrg	SD
OD1 Sample (mg)	12.54	12.74	0.20	195.60	76.69	75.07	1.43
OD2 Sample (mg)	12.53	12.73	0.20	201.30	74.52		
OD3 Sample (mg)	12.55	12.75	0.20	202.70	74.00		
Gastric Digestion						Avrg	SD
GD1 Sample (mg)	12.55	12.95	0.40	395.20	37.96	37.46	0.44
GD2 Sample (mg)	12.54	12.94	0.40	402.20	37.29		
GD3 Sample (mg)	12.56	12.96	0.40	404.00	37.13		
Intestinal Digestion						Avrg	SD
ID1 Sample (mg)	12.505	12.91	0.40	403.50	37.17	36.67	0.49
ID2 Sample (mg)	12.491	12.90	0.41	409.30	36.65		
ID3 Sample (mg)	12.512	12.93	0.41	414.40	36.20		

Figure S1: a. Representative MRM (Multiple Reaction Monitoring) chromatograms (Total amino acid profile); b. Glutamic acid; c. glutamine; d. Aspartic acid: e. asparagine.

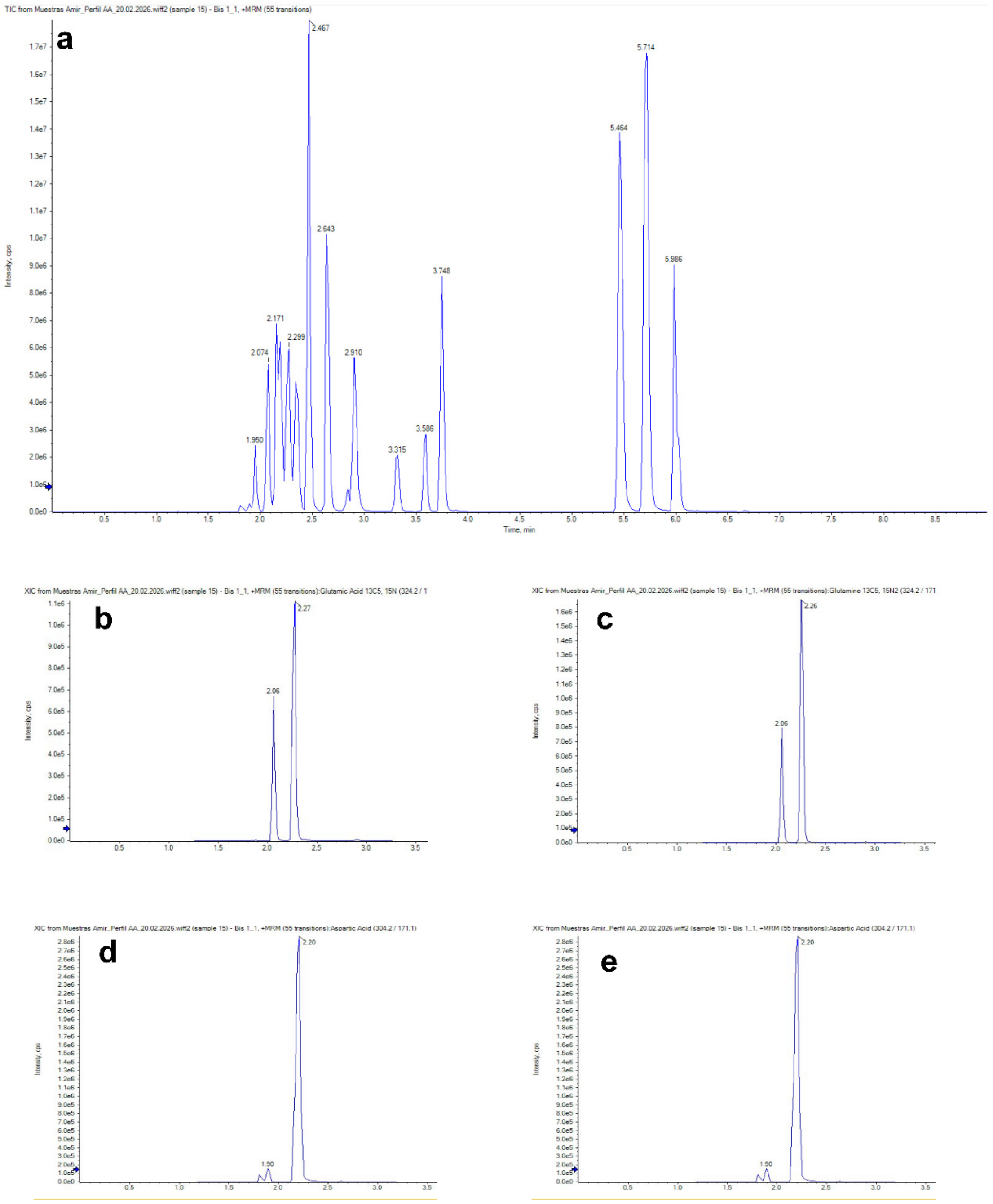

Figure S2: Representative GC-FID chromatogram of fatty acid methyl esters (FAMEs) from the lipidic fraction of *Bunium ferulaceum* seeds.

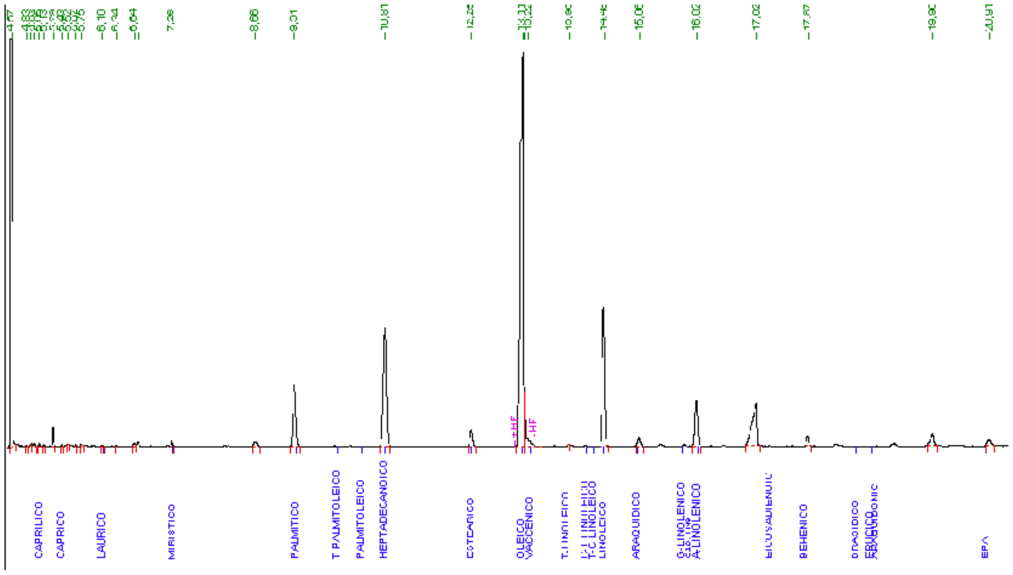

Supplement: Supplementary file 1 [file foods-15-03552-s001.zip › foods-4553469-supplementary.pdf]
